# New Insight into the Molecular Drug Target of Diabetic Nephropathy

**DOI:** 10.1155/2014/968681

**Published:** 2014-02-04

**Authors:** Vivian Soetikno, Wawaimuli Arozal, Melva Louisa, Rianto Setiabudy

**Affiliations:** Department of Pharmacology and Therapeutic, Faculty of Medicine, University of Indonesia, Salemba Raya 6, Jakarta 10430, Indonesia

## Abstract

Diabetic nephropathy (DN) lowered quality of life and shortened life expectancy amongst those affected. Evidence indicates interaction between advanced glycation end products (AGEs), activated protein kinase C (PKC) and angiotensin II exacerbate the progression of DN. Inhibitors of angiotensin-converting enzyme (ACEIs), renin angiotensin aldosterone system (RAAS), AGEs, and PKC have been tested for slowing down the progression of DN. The exact molecular drug targets that lead to the amelioration of renal injury in DN are not well understood. This review summarizes the potential therapeutic targets, based on putative mechanism in the progression of the disease.

## 1. Introduction

The number of people living with diabetes in the world is expected to double between 2000 and 2030. Population aging and urbanization increase the likelihood of this global health problem including the Asia-Pacific region. Indonesia as the fourth largest population country with a population of about 200 million shares a significant medical burden [1, 2]. This global increase in the prevalence of diabetes will inevitably lead to acceleration of micro- and macrovascular complications of diabetes. The important causative factor in the development of complications in patients with diabetes is hyperglycemia [3]. Diabetic nephropathy (DN) is the most common microvascular complication of diabetes mellitus. It is a leading cause of end-stage renal disease and a contributor to significant morbidity and mortality in patients with diabetes. About 20% of patients with either type 1 or type 2 diabetes develop nephropathy after many years of diabetes. There are many risk factors for the development of DN. They are uncontrolled hyperglycemia, hypertension, positive family history of nephropathy and hypertension, smoking, and racial or ethnic variation. In addition, sex hormones are an important determinant of DN. It has been reported that male gender is more prone to develop DN [4]. DN is characterized by albuminuria (≥300 mg/day), reduced glomerular filtration rate, and predisposition to chronic hyperglycemia during the prediabetic phase [5].

**Figure 1:**
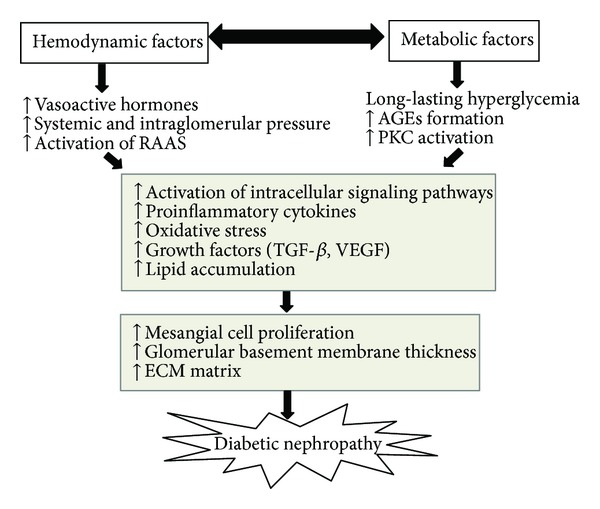
Schematic illustration of the interaction between hemodynamic and metabolic factors in the pathophysiology of diabetic nephropathy.

Over the last 20 years, theories have described how glucose promotes renal damage as illustrated in Figure 1. DN develops as a result of interactions between deleterious hemodynamic and metabolic factors. The interactions lead to the activation of intracellular signaling pathways and the activation of transcription factors triggered inflammatory mediators and growth factors release. These in turn mediated extracellular matrix (ECM) protein accumulation, vessel permeability alteration, and proteinuria [6, 7]. Previous treatment of DN focused on aggressive control of hyperglycemia and blood pressure. Currently, glucose-dependent pathways emerged as an important strategy to retard the progression of DN [6]. Several *in vitro* and *in vivo* studies have shown DN amelioration by managing the hyperglycemia-induced oxidative stress, inflammation, and lipid accumulation [8, 9]. Despite emerging strategies for retarding the progression of DN, the challenge for arresting the relentless progression of DN remains. In this review, the pharmacological targets of DN will be discussed, for example, vasoactive hormones, the biochemical processes of the advanced glycation end products (AGEs), protein kinase C (PKC), and AMP-activated protein kinases (AMPK) as well as novel pharmacological targets of DN, such as transcription factors nuclear factor erythroid 2-related factor 2 (Nrf2).

## 2. Approaches to the Treatment of DN

### 2.1. Hemodynamic Factors—Renin Angiotensin System (RAS)

The determinant of progression of DN involved not only systemic hypertension but also specific intrarenal changes, which can occur in the setting of normal blood pressure [10]. Intrarenal hemodynamic abnormalities including increased intraglomerular pressure, increased single nephron glomerular filtration rate, and preferential afferent versus efferent arteriolar vasodilation mediated progressive glomerular injury [11]. In recent years, RAS has been reported as a major mediator of renal injury. RAS activation by high glucose and mechanical stress can increase local formation of angiotensin II (Ang II) in the kidneys and causes many of pathophysiological changes associated with DN [12, 13]. Ang-II exerts complex hemodynamic and nonhemodynamic actions which contribute to DN, namely, induction of systemic vasoconstriction, increased glomerular arteriolar resistance and capillary pressure, increased glomerular capillary permeability, reduction in the filtration surface area, stimulation of ECM proteins, and renal proliferation [14–16]. It is also an important regulator of fluid and electrolyte balance, stimulates aldosterone production, activates the sympathetic nervous system, and increases sodium reabsorption [17, 18].

A new insight into the effects of Ang-II on DN postulated that the RAS has two axes: the ACE-Ang-II-AT1R axis and ACE-2-Ang-(1-7) *mas* R axis [14, 19, 20]. In contrast to the former axis, the latter axis induces vasodilation and antiproliferative, natriuretic, and diuretic effects and thus creates balance [21].

ACE-2 has a single catalytic domain and shares 40% homology with ACE. It has been shown to mediate the conversion of Ang-II to the heptapeptide Ang-(1-7), a peptide that antagonizes Ang-II signaling [22, 23]. ACE-2 also degrades Ang-I to Ang-(1-9) that is subsequently converted to Ang-(1-7) by ACE [24]. ACE-2 has been postulated to be an endogenous renoprotective enzyme based on the aforementioned properties [25]. ACE-2 has been localized to the podocyte in the kidneys. In experimental diabetes, there was a decrease in podocyte ACE-2 expression [26]. Transfection of human ACE-2 in streptozotocin (STZ)-induced diabetic mice's podocyte has been reported to have a protective effect against early development of albuminuria and to have a partial preservation effect of podocyte protein as well as podocyte number. The study also showed reduced glomerular histological injury and decreased kidney cortical TGF-*β*1 expression [27]. Thus, ACE-2 may represent a therapeutic target in the prevention and treatment of DN.

It has also been reported that Ang-(1-7) inhibits Ang-II mediated mitogen activated protein kinase (MAPK) activation by binding to the *mas* R [23], as well as inhibiting high glucose-induced p38 MAPK stimulation, which is believed to play a role in nephron injury associated with renal cell hypertrophy and fibrosis, which suggests that Ang-(1-7) provides protection against nephron injury by downregulating p38MAPK expression [28].

Based on the aforementioned studies, therapeutic blockade of the RAS with angiotensin-converting enzyme inhibitors (ACEIs) and/or AT1R blockers (ARBs) suppress the development and progression of DN in both type 1 and type 2 diabetic patients [29, 30]. Our study elaborated the experiment first and stated the confirmatory result; afterwards it demonstrated that treatment with olmesartan, an ARB, significantly attenuated Ang II mediated actions, such as oxidative stress and inflammatory reaction induction partially through the upregulation of ACE-2 and Ang-(1-7) *mas* R in STZ-induced diabetic mice [31]. Our group also has reported that telmisartan has beneficial effects on diabetic mice, mainly by its antifibrotic effects via activation of peroxisome proliferator activator (PPAR)-*γ* [32].

Albuminuria has long been accepted as the clinical hallmark of DN [33]. It signified glomerular damage and is considered as a nonhemodynamic promoter of disease progression in DN [34, 35]. Reducing proteinuria to less than 1 g/24 h has been added to the targets of glycemic control and lowered blood pressure goals in preventing progression of DN. Haller et al. (2011) demonstrated that olmesartan significantly attenuated the level of proteinuria in diabetic conditions [36]. Treatment with ARBs has been shown to have protective effects against the progression of DN by improving glomerular permeability, thus decreasing urine protein excretion levels [37, 38]. Experimental data shows that RAS blockade results in a change of nephrin expression, a slit-diaphragm protein, in diabetes [27, 39]. Nephrin mRNA expression, which is reduced in diabetes, can be preserved by ACEI [39]. These studies suggested that the RAS affects proteinuria in DN, partially by ameliorating diabetes-induced changes in nephrin synthesis.

Many studies reported high glucose and Ang-II stimulated collagen production by transforming growth factor (TGF)-*β*, a profibrotic cytokines [19, 40]. TGF-*β* expression is mainly increased in mesangial cells of diabetic human glomeruli [41], but increased TGF-*β* expression in glomerular endothelial cells has also been reported [42]. Inhibition of TGF-*β* results in prevention of fibrosis under experimental diabetic. Jiao et al. (2011) have shown that valsartan efficiently decreased oxidative stress in glomerular mesangial and epithelial cells cultured in high glucose conditions partially through the downregulation of TGF-*β* [40]. Chou et al. (2013) have demonstrated that high glucose decreases ACE2 via TGF-*β* receptor in NRK-52E cells. Furthermore, they showed that combination of TGF-*β* inhibition and ACE2-Ang-(1-7)-*mas* activation is useful for treating diabetic renal fibrosis [19]. Treatment with telmisartan, an ARB with PPAR-*γ* activity, in mice with hydronephrosis prevents renal atrophy and fibrosis. These beneficial actions of telmisartan were associated with a decrease in the renal expression of TGF-*β* and increased hepatocyte growth factor expression, a well-known antifibrotic factor [43].

### 2.2. AGEs—RAGE Modulation

AGEs are modifications of proteins or lipids that become nonenzymatically glycated and oxidized after contact with aldose sugars [44, 45]. In the early glycation and oxidation processes, the Schiff bases and Amadori products are formed and further glycation of proteins and lipids will cause molecular rearrangements that lead to the generation of AGEs [46]. AGEs are known to act as pathogenic substances that cause the activation of nuclear factor (NF)-*κ*B in tubular epithelial cells and TGF-*β*-Smad signaling in mesangial cells [47, 48]. It has also known that the activity of AGEs is mediated by their specific receptor, RAGE [44, 48]. Sasai et al. (2012) have found that AGEs can induce the expression of both plasminogen activator inhibitor-1 and tissue transglutaminase mRNA in tubular epithelial cells. Both gene products play an important role in the regulation of ECM degradations. They concluded that AGEs are powerful stimulants of renal epithelial cells, which stimulate the cells to recruit fibrosis-exacerbating macrophages [49].

Most AGE inhibitors have beneficial common downstream effects on oxidative stress in DN, including decreasing reactive oxygen species (ROS) production [50]. Indeed, interaction between AGEs and the RAGE are important for the development of renal impairments in diabetes. Previous studies demonstrated that the progression of DN was prevented by either RAGE deletion [51] or by RAGE-neutralizing antibodies [52]. In the diabetic RAGE-deficient mice, albuminuria, hyperfiltration, glomerulosclerosis, decreased renal mitochondrial ATP production, and excess generation of both mitochondrial and cytosolic superoxide were prevented. Glomerulosclerosis, tubulointerstitial expansion, and hyperfiltration were attenuated in diabetic mice treated with inhibitor of AGE accumulation, alagebrium chloride, which further emphasize the importance of AGE-RAGE blocking in amelioration of DN [53]. The inhibition of AGEs by alagebrium normalized the renal expression of RAGE and restored PKC activity to control level [54–56].

There was a direct interaction between AGEs and the RAS. ACEI and ARB significantly attenuated *in vitro *and *in vivo* production of AGEs such as pentosidine and *N* ^*ε*^-carboxymethyllysine (CML). The mechanism of action is through the reduction of reactive carbonyl and dicarbonyl compound (the precursor of AGE) production and reduction of oxidative metabolism [54]. These findings suggested that combination of AGE and RAS signaling pathway blockage may add synergistic effect in preventing DN progression.

### 2.3. PKC Inhibitors

PKC is a family of homologous serine/threonine kinases that catalyses numerous biochemical reactions critical to cellular functions and has an important role in the pathophysiology of diabetes complications, such as ECM basement membrane production, growth factors release, and albuminuria. The PKC family can be divided into three isoforms, namely, classical (*α*, *β*I, *β*II, *γ*), novel (*δ*, *ε*, *η*, *θ*), and atypical (*ζ*, **ι**/*λ*) [57].

Renal injury in diabetes is known to be associated with the activation of the diacylglycerol (DAG)-PKC pathway. The major source of DAG is *de novo* synthesis from glycolytic intermediates. There is an elevation of DAG production in diabetes which subsequently increases the PKC activity. PKC activity was increased in mesangial cells or in glomeruli and proximal tubule cells under high glucose exposure [57–59]. Previous study has demonstrated that high glucose-induced oxidative stress and PKC activation cause cellular hypertrophy which resulted in renal proximal tubule enlargement. The addition of staurosporine, inhibitor of PKC, blocked a high glucose-induced increase of TGF-*β*1 [60]. It has been demonstrated that activation of the PKC-*β* isoform contributes to high glucose-mediated renal hypertrophy and ECM expansion [61, 62]. High glucose-induced albuminuria is mediated partially by activation of the PKC-*α* isoform. PKC-*α* isoform regulates vascular endothelial growth factor (VEGF) as well as nephrin expression [63, 64].

We have also previously demonstrated that diabetes induced upregulation of VEGF and TGF-*β*1 expression in STZ-induced DN. This is mediated partially by the activation of PKC-*α* and -*β*. The activations of both PKC were ameliorated significantly by curcumin treatment, a powerful antioxidant [65]. Menne et al. (2013) have revealed that blockade of PKC-*α* and -*β* isoforms resulted beneficial effects on albuminuria and on the development of renal hypertrophy under diabetic conditions [66]. In contrary, a clinical study with selective PKC-*β* inhibitor, ruboxistaurin, for patients with diabetic retinopathy showed loss of kidney function, irrespective of placebo or ruboxistaurin treatment, during 3 years study [67]. Even though the results from *in vivo* studies showed to be promising, the dual inhibitor of classical PKC needs further study in clinical trial.

### 2.4. Activation of AMPK

AMPK was first identified as a kinase for hydroxymethylglutaryl-CoA reductase (HMG-CoA) and acetyl-CoA carboxylase (ACC), key regulatory enzymes of steroid and fatty acid synthesis, respectively [68]. The AMPK heterotrimer consists of a catalytic *α*-subunit and regulatory *β*- and *γ*-subunits. There are multiple isoforms for each subunit (*α*1 and *α*2, *β*1 and *β*2, and *γ*1, *γ*2, and *γ*3) with tissue-specific distribution. In kidneys, *α*1 isoform is ubiquitously expressed, whereas the *α*2 isoform has its highest levels of expression in liver, heart, and skeletal muscle [68, 69]. The enzymatic activity of AMPK is dependent on phosphorylation of Thr172 of the *α*-subunit [68]. Various metabolic stresses which caused an increase in AMP : ATP ratio mainly activate AMPK. AMPK inhibits energy-consuming reactions such as synthesis of fatty acids and sterols and activates ATP-generating processes such as fatty acid oxidation [70].

Glucose and lipid metabolism have been linked together for decades. The most common cause of progressive kidney disease is DN with microalbuminuria as its earliest manifestation. Acquired lipodystrophy which leads to ectopic lipid deposition (i.e., lipid deposition in nonadipose tissues) has been documented in the chronic kidney disease (CKD). Adipocyte differentiation-regulated protein (ADRP or adipophilin in humans) is a 50-kDa fatty acid binding protein that is activated when preadipocytes differentiate into mature adipocytes. It will fill the cytoplasm with lipid storage droplets [71].

CKD induces dyslipidemia with increased plasma triglycerides; thus, lipid-lowering strategies have shown beneficial effect [72–74]. Studies have shown that the accumulation of lipids (triglycerides and cholesterol) in the glomerular and tubule-interstitial cells were associated with increased expression of sterol regulatory element binding protein (SREBP)-1, a transcription factor which plays a key role in lipogenesis and its related genes (ACC, acetyl-CoA Carboxylase, and FAS, fatty acid synthase). These changes were associated with significant glomerulosclerosis and proteinuria [75, 76]. Our study confirmed that STZ-induced DN exhibited ectopic lipid distribution mainly in kidney glomeruli. There is also association with changes in AMPK activity and SREBP-1c expression. These abnormalities were related to activation of oxidative stress and inflammation [77]. Besides functions as a regulator of lipid metabolism, AMPK is also known to play a key role in cell hypertrophy.

Previous study has shown that diabetes-induced renal hypertrophy was inhibited by metformin or 5-aminoimidazole-4-carboxamide-1*β*-riboside (AICAR) (both are well-known AMPK activators). Both treatments prevented the reduction in AMPK phosphorylation in renal cortex in diabetic rat, which suggested that reduction AMPK activity thus contributed to diabetes-induced renal hypertrophy in rats [78]. The influence of mammalian target of rapamycin (mTOR) on renal tubular cells lipid metabolism in diabetes was reported. Brown et al. (2007) have reported that mTOR signaling plays a major role in regulation of SREBP-1c expression in hepatocytes, which in turn activates ACC, FAS, and stearoyl-CoA desaturase-1 and -2 (SCD) enzymes involved in lipogenesis [79]. The expression of SREBP-1 and ADRP, a classical marker of lipid droplet, was upregulated, as well as the cellular triglyceride was increased in the renal tubular cells of diabetic rats. Rapamycin, the classical mTOR activity inhibitor, decreased mTOR activity and prevented high glucose-induced SREBP-1 upregulation and cellular lipid droplet which suggested that mTOR inhibition might be the promising drug molecular target for treating renal lipid deposit of DN [80].

### 2.5. Keap1-Nrf2 Signaling Pathway

Nrf2 is a transcription factor that mediates a wide range of adaptive responses to intrinsic and extrinsic cellular stresses. Nrf2 influences many signaling cascades for detoxifying harmful substances and maintains cellular redox homeostasis [81]. Under basal conditions, Nrf2 is sequestered in the cytosol by its inhibitor protein, Keap1 homodimer, which facilitates Nrf2's ubiquitination and proteasomal degradation via the proteasome. Under cellular insult such as chemical, oxidative, and electrophilic stresses or in the presence of Nrf2 activating compounds, this degradation is hindered and Nrf2 translocates to the nucleus and binds to the antioxidant response element. This in turn induces the expression of cellular defensive genes, such as antioxidant enzymes. Antioxidant enzymes then increased the levels of glutathione synthesis and regeneration, stimulated nicotinamide adenine dinucleotide phosphate (NADPH) synthesis; enhanced toxin efflux via the multidrug-response transporters; enhanced the recognition, repair, and removal of damages proteins; regulated expression of other transcription factors, growth factor and its receptors as well as molecular chaperones; and reduced reactive compounds to less toxic intermediates [82].

Recent findings showed that Nrf2 is functionally involved in the synthesis and metabolism of fatty acids [83], inflammatory processes via attenuation of NF-*κ*B pathway [84], and fibrosis through modulation of TGF-*β* [85, 86]. High glucose-induced renal damage is associated with excessive production of ROS as reported in several *in vitro* and *in vivo* studies [87, 88]. Oxidative stress plays a key role in the pathogenesis of vascular complications, both micro- and macrovascular in diabetes, while the role of oxidative stress in diabetes is questioned by the obscure results of intervention studies with antioxidants [89].

The role of Nrf2 in protecting against diabetic vascular diseases has recently been reported. Activation of Nrf2 by sulforaphane suppressed hyperglycemia-induced ROS and metabolic dysfunction in human microvascular endothelial cells [90]. Using Nrf2^−/−^ and Nrf2^+/+^ mice, Jiang et al. (2010) demonstrated that Nrf2 conferred protection against hyperglycemia-induced renal damage. They showed that Nrf2 negatively regulates TGF-*β*1 and its downstream effectors, such as ECM production [91]. Zheng et al. (2011) reported a beneficial role of Nrf2 activator such as sulforaphane and cinnamic aldehyde against DN using STZ-induced diabetes in Nrf2 knock-out mice. They showed that sulforaphane and cinnamic aldehyde significantly reduced common metabolic disorder associated with diabetes, including hyperglycemia, polydipsia, polyuria, and weight loss. In the kidney tissues, both treatments retarded pathological characteristics of DN such as oxidative damage, albuminuria, renal hypertrophy, ECM deposition, and thickening of basement membranes. This suggested sulforaphane and cinnamic aldehyde function through specific activation of the Nrf2 pathway [92]. Recently, several attempts have been made at using Nrf2 activators as treatment for diabetes. Treatment with bardoxolone for type 2 diabetes patients with CKD showed significant improvements in the estimated glomerular filtration rate with mild to moderate side effects [93, 94].

Treatment with curcumin, a natural phytocompound that acts as an Nrf2 activator, significantly attenuated high fat diet-fed mice-induced muscular oxidative stress by activating Nrf2 function. This is a novel mechanism for improving glucose intolerance [95]. Our study showed that treatment with curcumin significantly ameliorated CKD-induced oxidative stress and inflammation in the remnant kidney tissue, partially through the activation of Nrf2 [96]. The discovery of a number of both natural and synthetic compounds which can be able to induced Nrf2 has emerged over the past decade. The pharmacological activation of Nrf2 by various compounds namely allyl sulfides, dithiolethiones, flavonoids, isothiocyanates, polyphenols and triterpenoids. These compounds have been proposed for prevention of a number of oxidative stress-related diseases [97].

### 2.6. Oxidative Stress and NADPH Oxidase

It has been postulated that hyperglycemia is the initiating cause of the diabetic tissue damage. Both mesangial cells and endothelial cells are the two most cell types in our body which are more prone to high levels of glucose due to the fact that the glucose transport rate of those cells does not decline rapidly as a result of hyperglycemia [98]. In the presence of increased glucose, endothelial cells generate an extra of ROS, particularly superoxide anion [99]. Several pathways can be considered as candidates for oxygen- free radical formation in cells, such as NADPH oxidase, xanthine oxidase, and microsomal enzymes. NADPH oxidases may be considered as the most important enzymes whose primary function is regulation of ROS production.

The NADPH oxidase complex plays an essential role in nonspecific host defense against microbial organisms and was originally identified in phagocytes. This phagocytic enzyme is normally quiescent, but it becomes activated during the oxidative process and generates large amount of superoxide anion [100].

Broadly comparable NADPH oxidase enzymes have been revealed in numerous nonphagocytic cell types, including endothelial cells, cardiomyocytes, and fibroblasts [101]. The molecular composition of these nonphagocytic enzymes is homologues of the gp91phox catalytic subunit. These homologues are designated Nox. Nox4 was cloned from the kidney and found to be highly expressed in this organ; the expression was reported to be increased in a diabetic state, which induces renal hypertrophy and increases fibronectin expression [62, 102]. Impairment of Nox4 activity using knock-down strategy in a diabetic condition reduced high glucose-induced podocyte apoptosis, which suggests that Nox4 plays a key role in diabetes-induced albuminuria [103]. Sedeek et al. (2013) have suggested that a Nox4 inhibitor prevents the development of nephropathy in *db/db* mice independently of improved glucose control [104]. Eid et al. (2013) lately demonstrated that rapamycin decreased the expression of Nox4 and hyperglycemia-induced podocyte apoptosis [105]. Thus, both mTOR and NADPH oxidase inhibitor may be potential drug target in preventing complication and progression of DN.

**Figure 2:**
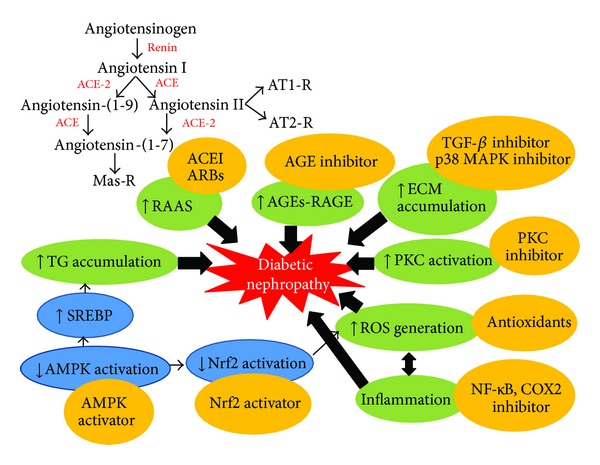
Current drug targets in diabetic nephropathy. Interrelated derangements in cellular hemodynamic, metabolic, oxidative stress, inflammation; lipid dysfunction, and extracellular matrix accumulation occur in diabetes and lead to diabetic nephropathy. These derangements provide the targets for the development of mechanism based drugs.

Based on the findings from the above studies, it can be concluded that numerous factors are implicated in the development and progression of DN. The disruption of RAS remains the gold standard therapy to prevent the progression of DN. In addition, strict control of hyperglycemia, blood pressure control, and hyperlipidemia treatment are critical factor for optimizing renal functions in diabetes. Besides hemodynamic and metabolic abnormalities, there is a broad range of abnormalities associated with oxidative stress, inflammation and lipid accumulation. These have emerged in the new perspective of DN pathophysiology and potential therapeutic intervention of DN (Figure 2). Hence, it shows that all of the molecular signaling pathways were important and interrelated in the development and progression of DN.

## 3. Conclusion

DN continues to be a major complication of type 1 and type 2 diabetes and represents the major cause of end-stage renal disease. New therapies for DN are urgently needed. There are multiple signaling pathways involved in the progressive development of DN. It is probable that further efforts in the discovery newly identified compounds or development of new synthetic drugs required multi-targeted therapies will reach the ultimate goal, that is, full prevention the progression of DN might be in sight.
